# Analysis of agreement among definitions of metabolic syndrome in nondiabetic Turkish adults: a methodological study

**DOI:** 10.1186/1471-2458-7-353

**Published:** 2007-12-19

**Authors:** Ahmet Selcuk Can, Thomas P Bersot

**Affiliations:** 1 Department of Medicine, Istanbul Science University, Faculty of Medicine, Esentepe, Sisli, Istanbul, Turkey; 2 Gladstone Institute of Cardiovascular Disease and the University of California, San Francisco, California, USA

## Abstract

**Background:**

We aimed to explore the agreement among World Health Organization (WHO), European Group for the Study of Insulin Resistance (EGIR), National Cholesterol Education Program (NCEP), American College of Endocrinology (ACE), and International Diabetes Federation (IDF) definitions of the metabolic syndrome.

**Methods:**

1568 subjects (532 men, 1036 women, mean age 45 and standard deviation (SD) 13 years) were evaluated in this cross-sectional, methodological study. Cardiometabolic risk factors were determined. Insulin sensitivity was calculated by HOMA-IR. Agreement among definitions was determined by the kappa statistic. ANOVA and post hoc Tukey's test were used to compare multiple groups.

**Results:**

The agreement between WHO and EGIR definitions was very good (kappa: 0.83). The agreement between NCEP, ACE, and IDF definitions was substantial to very good (kappa: 0.77–0.84). The agreement between NCEP or ACE or IDF and WHO or EGIR definitions was fair (kappa: 0.32–0.37). The age and sex adjusted prevalence of metabolic syndrome was 38% by NCEP, 42% by ACE and IDF, 20% by EGIR and 19% by WHO definition. The evaluated definitions were dichotomized after analysis of design, agreement and prevalence: insulin measurement requiring definitions (WHO and EGIR) and definitions not requiring insulin measurement (NCEP, ACE, IDF). One definition was selected from each set for comparison. WHO-defined subjects were more insulin resistant than subjects without the metabolic syndrome (mean and SD for log HOMA-IR, 0.53 ± 0.14 vs. 0.07 ± 0.23, respectively, p < 0.05) and had higher Framingham risk scores (mean and SD, 2.99 ± 4.64% vs. 1.10 ± 1.87%, respectively, p < 0.05). The additional subjects identified by IDF definition, but not by WHO definition also had more insulin resistance and higher Framingham risk scores than subjects without the metabolic syndrome (mean and SD, log HOMA-IR 0.18 ± 0.18 vs. 0.07 ± 0.23, p < 0.05 and Framingham risk score 2.93 ± 4.54% vs. 1.10 ± 1.87%, p < 0.05). The IDF-identified additional subjects had similar Framingham risk scores as WHO-identified subjects (p > 0.05), but lower log HOMA-IR values (p < 0.05).

**Conclusion:**

The metabolic syndrome definitions that do not require measurement of insulin levels (NCEP, ACE and IDF) identify twice more patients with insulin resistance and increased Framingham risk scores and are more useful than the definitions that require measurement of insulin levels (WHO and EGIR).

## Background

The term "metabolic syndrome" describes clustering of cardiovascular disease (CVD) risk factors of metabolic origin [1,2]. A two-fold increase in the risk of CVD and a five-fold increase in the risk of type 2 diabetes mellitus accompany the metabolic syndrome [3-8]. World Health Organization (WHO) was the first to propose criteria for diagnosis of the metabolic syndrome [9], followed by the European Group for the Study of Insulin Resistance (EGIR) [10], National Cholesterol Education Program (NCEP) Adult Treatment Panel III [1], American College of Endocrinology (ACE) [11], and International Diabetes Federation (IDF) [12]. Although these organizations proposed to measure the same components, they suggested different combinations and different cut-off points. In WHO and EGIR definitions, the presence of insulin resistance was the starting point. In IDF definition, central obesity was the prerequisite of the metabolic syndrome. Components of the metabolic syndrome were selected by these organizations, because they tend to cluster commonly in insulin resistant individuals who are at increased risk of CVD, beyond the risk implicated by classical CVD risk factors, like elevated low density lipoprotein cholesterol (LDL-C) levels [5,8,13-15]. Therefore, the metabolic syndrome has been assigned as a secondary target for intervention by NCEP Adult Treatment Panel III. So far information on the agreement among all of the five definitions of the metabolic syndrome is limited, especially data on IDF definition is recently accumulating [5,16-24]. The aim of our study was to assess the agreement among various definitions of the metabolic syndrome and to explore the differences in anthropometric and metabolic variables among WHO, EGIR, NCEP, ACE and IDF definition-identified subjects.

## Methods

This was a methodological analysis based on data derived from the Turkish Heart Study, a cross-sectional epidemiological survey of CVD risk factors in Turkish adults [25]. Information about past medical history, family history, demographic characteristics, socioeconomic status, physical activity level, smoking, and drinking habits was obtained with a physician interview. Subjects were classified as low income, if the monthly household income was less than $500; as low education level if they were illiterate, literate only or had five years or less of elementary school education. Subjects who report less than one hour of physical exercise per week were classified as sedentary. Subjects who consumed at least one alcoholic beverage per month were assigned to the drinker category. Family history was obtained for first degree relatives. Body mass index (BMI) was calculated as weight in kilograms over squared height in meters. Height was measured to within 0.5 cm with a measuring stick, weight to within 0.1 kg with a digital scale, waist and hip circumference to the nearest 0.5 cm. Waist circumference was measured at the midway between lower margin of the rib cage and the superior iliac crest during mild expiration. Hip circumference was measured at the greater trochanteric level with a measuring tape. All measurements were taken with shoes removed and with participants wearing light clothing. Blood pressure was measured on the right arm with an automated sphygmomanometer (Omron automatic blood pressure monitor with IntelliSense^®^, Bannockburn, Illinois, USA) after fifteen minute of rest with the subject in the sitting position. The mean of two recordings, five minutes apart was recorded.

Subjects were invited to the study from the offices of local governors by our staff and with posted fliers. No financial incentive was paid to the participants. More explanation about subject recruitment was provided in Additional file 1. 1700 subjects participated to the study in 2003. One hundred and nineteen subjects (7%) with past history of or newly diagnosed diabetes mellitus were excluded from this analysis because ACE and EGIR exempted diabetics from their definitions. Eleven subjects below the age of 20 and two pregnant subjects were also excluded. There are 1568 subjects, 532 men (34%) and 1036 women (66%) in this analysis. 1050 participants (67%) were from Istanbul, an urban area and 518 participants (33%) were from Kayseri, a rural area. Six percent (*n *= 95) of the subjects had a history of self-reported cardiovascular disease. Fifty-four subjects (3%) were taking lipid lowering medications. Forty-five subjects were taking statins, three subjects fibrates, six subjects omega-3 fatty acids and none of the subjects were taking niacin or resins. Two hundred and thirty-six subjects (15%) were taking antihypertensive agents. General characteristics of the study subjects are given in Table 1. The study procedures were approved by the Committee on Human Research of the University of California, San Francisco and permission to conduct the study was granted by the Ministry of Health, Republic of Turkey. All subjects signed written informed consent.

**Table 1:** General characteristics of the subjects.

	All	Men	Women
*n*	1568	532	1036
Age (years)*	45 ± 13	45 ± 13	45 ± 13
Low income†	72%	59%	78%
Low education†	60%	43%	69%
Sedentary†	73%	65%	77%
Current smoker†	26%	40%	19%
Drinker†	22%	42%	11%
BMI (kg/m^2^)*	29 ± 5	28 ± 4	30 ± 5
Waist (cm)*	94.6 ± 12.3	99.6 ± 10.2	92.1 ± 12.5
Glucose (mmol/l)*	5.0 ± 0.6	5.1 ± 0.5	5.0 ± 0.6
Insulin (pmol/l)‡	51.7 (35.9, 73.2)	52.4 (35.9, 78.2)	50.9 (35.9, 71.8)
HOMA-IR‡	1.57 (1.08, 2.32)	1.65 (1.12, 2.47)	1.53 (1.05, 2.30)
TC (mmol/l)*	4.75 ± 1.02	4.70 ± 0.93	4.77 ± 1.06
HDL-C (mmol/l)*	1.15 ± 0.31	1.00 ± 0.25	1.23 ± 0.31
LDL-C (mmol/l)*	2.92 ± 0.88	2.91 ± 0.82	2.93 ± 0.91
Triglyceride (mmol/l)‡	1.26 (0.91, 1.77)	1.45 (1.01, 2.09)	1.19 (0.86, 1.65)

Please see list of abbreviations used. *mean ± SD are given for normally distributed variables, †percentage values are given for categorical variables ‡median (25^th ^and 75^th ^percentile values) are given for variables with a skewed distribution

Blood was collected after a 10-hour fast. Kits from Boehringer-Mannheim (Mannheim, Germany) were used for lipid and glucose analyses. A multichannel analyzer (H 917, Hitachi, Tokyo, Japan) was used for colorimetric enzymatic determinations of cholesterol (kit: Monotest Cholesterol with cholesterol esterase, cholesterol oxidase and peroxidase (CHOD-PAP)), triglycerides (kit: Peridochrom Triglyceride with glycerol phosphate oxidase and peroxidase (GPO-PAP)) and glucose (kit: Glucose, glucose oxidase and peroxidase (GOD-PAP)). LDL cholesterol (LDL-C) was calculated by the Friedewald equation [26] for participants with triglyceride levels < 500 mg/dl. A homogenous assay for measuring HDL levels was used. Fasting insulin levels were measured with an electrochemiluminescence immunoassay (Roche Elecsys 2010, Roche Diagnostics, kit: Insulin). Insulin sensitivity was estimated with Homeostasis Model Assessment (HOMA-IR) equation. HOMA-IR equals fasting serum insulin (μU/ml) times fasting plasma glucose (mmol/l) divided by 22.5 [27]. Biochemical analyses were performed at the American Hospital Clinical Laboratory in Istanbul, as it is certified as a reference laboratory by the Centers for Disease Control (Atlanta, Georgia, USA).

The WHO, EGIR, NCEP, ACE, and IDF criteria for metabolic syndrome is outlined in Table 2. American Heart Association and National Heart Lung and Blood Institute modified NCEP criteria by decreasing the glucose cut-off from 6.1 to 5.6 mmol/l and assigned patients on drug therapy for hypertension, hypertriglyceridemia, and low HDL-C levels to positive components [28]. We used the modified NCEP criteria. EGIR defines insulin resistance, as plasma insulin levels above 75^th ^percentile [10]. The 75^th ^percentile value of insulin distribution in our study sample of nondiabetic individuals was 73.2 pmol/l (10.2 μU/ml). Similar to EGIR, WHO designates insulin resistance as a prerequisite for the diagnosis of the metabolic syndrome. WHO defines insulin resistance as "under euglycemic hyperinsulinemic conditions, glucose uptake below the lowest quartile for background population under investigation" [9]. As euglycemic hyperinsulinemic clamp studies are not routinely performed in clinical practice and they are time consuming and costly, we used HOMA-IR to define insulin resistance. A similar modification was used in previous studies [4,7]. We designated the subjects in the upper quartile of the HOMA-IR distribution as insulin resistant. The 75^th ^percentile HOMA-IR value in nondiabetic subjects was 2.32 in this study. As urinary microalbumin excretion was not ascertained in this study, it was not counted as a component in the WHO-defined metabolic syndrome. Oral glucose tolerance test was not performed and postprandial glucose results were not included in the glucose component of ACE and WHO definitions.

**Table 2:** Criteria for clinical diagnosis of the metabolic syndrome.

	WHO	EGIR	NCEP	ACE	IDF
Prerequisite	DM, IFG, IGT, IR	insulin in top 25%	None	high risk*	WC ≥ 94 cm (men), WC ≥ 80 cm (women)†
No of criteria	and ≥ 2 of	and ≥ 2 of	≥ 3 of	and ≥ 2 of	and ≥ 2 of
Obesity	BMI > 30 and/or WHR > 0.9 (men), WHR > 0.85 (women)	WC ≥ 94 cm (men), WC ≥ 80 cm (women)	WC ≥ 102 cm (men), WC ≥ 88 cm (women)		
BP (mmHg)	≥ 140/90	≥ 140/90‡	≥ 130/85‡	>130/85	≥ 130/85‡
HDL-C (mmol/l)	<0.9 (men), <1.0 (women)§ or	<1.0‡§ or	<1.0 (men), <1.3 (women)‡	<1.0 (men), <1.3 (women)	<1.0 (men), <1.3 (women)‡
TG (mmol/l)	≥ 1.7	>2.0‡	≥ 1.7‡	>1.7	>1.7‡
Glucose (mmol/l)	≥ 6.1, IGT	≥ 6.1 ||	≥ 5.6‡	≥ 6.1, IGT ||	≥ 5.6‡

Please see list of abbreviations used. *high risk: diagnosis of CVD, hypertension, polycystic ovary syndrome, nonalcoholic fatty liver disease, or acanthosis nigricans; family history of type 2 diabetes, hypertension, or CVD; history of gestational diabetes or glucose intolerance; Non-Caucasian ethnicity, sedentary lifestyle, BMI > 25 kg/m^2 ^or WC > 94 cm (men), >80 cm (women); and age >40 years [11]. †cut-off values differ according to ethnic origin [12]. ‡or treated for this abnormality. §lipid criterion consists of elevated triglycerides and/or low HDL in WHO and EGIR definitions and is counted as one component. || excludes patients with diabetes.

The Statistical Package for Social Sciences software, version 15.0 (SPSS Inc., Chicago, Illinois, USA) was used for statistical analyses. Data were summarized by calculating mean ± standard deviation for quantitative variables and percentages for categorical variables. The agreement between five definitions of the metabolic syndrome was determined by the kappa statistic (κ). The level of agreement is considered poor with κ ≤ 0.20, fair with κ = 0.21 to 0.40, moderate with κ = 0.41 to 0.60, substantial with κ = 0.61 to 0.80, and very good with κ > 0.80 [29]. Age and sex adjusted prevalence of the metabolic syndrome was calculated separately for each definition. Information from the Republic of Turkey, Ministry of Interior Affairs, Office of Population and Citizenship 2003 household survey was used to adjust the prevalence rates by the direct method [30]. Ten year risk of coronary heart disease was calculated from Framingham risk tables [1]. One-way ANOVA was performed in order to analyze mean differences among multiple groups. When significant differences were found, post hoc Tukey's test was performed for identifying the source of these differences.

## Results

The age and sex adjusted prevalence of the metabolic syndrome was 38% by NCEP, 42% by ACE and IDF, 20% by EGIR, and 19% by WHO definition. The age and sex adjusted prevalence of each metabolic syndrome component was as follows: waist circumference according to NCEP criteria: 49%, waist circumference according to IDF criteria: 73%, elevated triglycerides (NCEP): 28%, low HDL-C (NCEP): 61%, elevated blood pressure (NCEP): 56%, elevated glucose (NCEP, excluding diabetes): 15%. The agreement among all of the five published definitions of the metabolic syndrome is shown in Table 3. The agreement among NCEP, ACE, and IDF definitions was substantial to very good. The agreement between WHO and EGIR definitions was also very good. The agreement between NCEP or ACE or IDF and WHO or EGIR definitions was fair and κ value ranged from 0.32 to 0.37. The agreement among metabolic syndrome definitions was further assessed according to geographic area and gender (Table 3a and Table 3b in Additional file 2). Forty-six percent (*n *= 722) of our study sample was categorized as not having the metabolic syndrome and 14% (*n *= 224) as having the metabolic syndrome, concordantly according to all five definitions. Seventy-seven percent (*n *= 1203) of the subjects was categorized as not having and 18% (*n *= 279) as having the metabolic syndrome for both WHO and EGIR definitions. Forty-nine percent (*n *= 766) of the participants was categorized as not having the metabolic syndrome and 37% (*n *= 575) as having the metabolic syndrome concordantly by NCEP, ACE and IDF definitions.

**Table 3:** Agreement between each definition of the metabolic syndrome.

	NCEP	IDF	ACE	WHO	EGIR
NCEP	*	0.84	0.77	0.37	0.37
IDF		*	0.81	0.33	0.35
ACE			*	0.32	0.34
WHO				*	0.83
EGIR					*

Please see list of abbreviations used. *n *= 1568

Anthropometrics, biochemical data and Framingham risk scores of subjects categorized as metabolic syndrome according to each definition are provided in Table 4. All of the WHO definition, 92% of EGIR, 44% of NCEP, 40% of ACE, and 42% of IDF definition-identified subjects had insulin resistance, as defined being in the top quartile of HOMA-IR distribution in our sample. The frequency of insulin resistance in subjects free of the metabolic syndrome according to any of the definitions was less than 14%. Table 4 shows that each definition of the metabolic syndrome identifies a subset of individuals from the study population with almost the same characteristics. The most striking differences are in the prevalence rates, fasting insulin levels and HOMA-IR values. Our data (Table 3 and 4) indicate that definitions of the metabolic syndrome can be dichotomized into two sets: the first set, NCEP, ACE and IDF, and the second set, WHO and EGIR definitions. The most prominent feature of the first set is that NCEP, ACE and IDF definitions do not require the measurement of insulin levels in their criteria, whereas the second set, WHO and EGIR definitions do require the assessment of insulin sensitivity or insulin levels. Prevalence estimates show that the first set of definitions categorizes twice more subjects as metabolic syndrome cases than the second set. In view of these results, we wished to assess in which metabolic variables the surplus subjects, differed from subjects free of the metabolic syndrome or from subjects identified by an insulin measurement requiring definition. One definition from each set was selected for this purpose, for example WHO and IDF definitions. Subjects who do not have the metabolic syndrome according to WHO definition constituted the first group. The second group was subjects with the metabolic syndrome according to WHO definition including subjects who concordantly satisfy the IDF definition at the same time. The third group was subjects with surplus metabolic syndrome, indicating those categorized as metabolic syndrome cases only by IDF definition, but not by WHO definition (see Table 5). Subjects with the metabolic syndrome according to WHO definition had significantly higher insulin levels and Framingham risk scores than subjects without the metabolic syndrome. Surplus subjects classified as metabolic syndrome by IDF but not by WHO definition also had significantly higher fasting insulin levels than subjects without the metabolic syndrome. The Framingham risk scores of surplus-metabolic syndrome subjects were also significantly higher than subjects without the metabolic syndrome. Subjects with IDF-defined surplus metabolic syndrome had slightly but significantly lower BMI, waist circumference, diastolic blood pressure, insulin, HOMA and triglyceride values than subjects with WHO-defined metabolic syndrome. There were no differences in the Framingham risk scores between WHO- and surplus IDF-defined subjects with the metabolic syndrome. We replaced EGIR for WHO definition and NCEP and ACE for IDF definition and repeated the comparison in the same manner. These comparisons are provided in tables 6-10 in Additional files 3, 4, 5, 6, 7. Subjects with the metabolic syndrome as defined either by WHO and EGIR criteria had an adverse CVD risk factor profile compared with subjects without the metabolic syndrome. Surplus metabolic syndrome subjects defined by IDF, NCEP or ACE definition had higher BMI, waist circumference, SBP, DBP; glucose, insulin, HOMA-IR, TC, and TG values and lower HDL-C values than subjects without the metabolic syndrome. Surplus metabolic syndrome (IDF-, NCEP-, ACE-defined) cases had slightly, but significantly lower BMI, waist circumference, insulin, HOMA-IR and TG values than subjects identified by an insulin measurement requiring definition (WHO or EGIR). Except for minimal differences in systolic blood pressure (up to 3 mmHg), total cholesterol level (up to 0.15 mmol/l) and log triglycerides (up to 0.05), surplus metabolic syndrome cases had the same degree of impairment in measured variables whether they were categorized by IDF, NCEP or ACE definition (tables 6-10 in Additional files 3, 4, 5, 6, 7).

**Table 4:** Anthropometric and biochemical data of subjects categorized as metabolic syndrome according to each studied definition.

	WHO-MS	EGIR-MS	NCEP-MS	ACE-MS	IDF-MS
Age (years)*	47 ± 12	46 ± 12	48 ± 12	48 ± 12	48 ± 12
BMI (kg/m^2^)*	33 ± 5	33 ± 5	32 ± 4	31 ± 5	32 ± 5
SBP (mmHg)*	145 ± 22	144 ± 23	145 ± 24	143 ± 24	144 ± 25
DBP (mmHg)*	93 ± 12	92 ± 12	92 ± 12	91 ± 12	91 ± 12
Glucose (mmol/l)*	5.4 ± 0.6	5.3 ± 0.6	5.2 ± 0.6	5.1 ± 0.6	5.2 ± 0.6
Insulin (pmol/l)†	96.1 (81.6, 119.1)	96.1 (82.3, 118.6)	66.0 (47.4, 92.6)	63.9 (44.5, 90.4)	64.6 (45.9, 90.6)
HOMA-IR†	3.14 (2.64, 4.03)	3.12 (2.65, 4.03)	2.13 (1.48, 3.00)	2.00 (1.36, 2.89)	2.08 (1.44, 2.95)
Framingham risk score*	2.99 ± 4.64	2.79 ± 4.45	3.22 ± 4.81	3.08 ± 4.71	3.04 ± 4.67
Women:					
Frequency‡	18%	19%	43%	44%	48%
Waist (cm)*	102.8 ± 9.7	101.3 ± 10.1	99.6 ± 9.7	97.6 ± 10.9	98.2 ± 10.2
TC (mmol/l)*	4.99 ± 1.13	4.94 ± 1.13	4.95 ± 1.06	4.92 ± 1.10	4.91 ± 1.05
HDL-C (mmol/l)*	1.07 ± 0.26	1.06 ± 0.27	1.06 ± 0.23	1.05 ± 0.22	1.07 ± 0.23
LDL-C (mmol/l)*	3.06 ± 0.97	3.04 ± 0.96	3.09 ± 0.91	3.06 ± 0.95	3.05 ± 0.89
TG (mmol/l)†	1.72 (1.30, 2.34)	1.70 (1.26, 2.29)	1.59 (1.19, 2.13)	1.67 (1.21, 2.18)	1.57 (1.13, 2.07)
Men:					
Frequency‡	23%	25%	41%	48%	46%
Waist (cm)*	107.0 ± 8.3	106.9 ± 8.12	105.3 ± 8.5	102.9 ± 9.1	105.2 ± 7.1
TC (mmol/l)*	4.89 ± 0.91	4.85 ± 0.90	4.87 ± 0.84	4.78 ± 0.88	4.82 ± 0.90
HDL-C (mmol/l)*	0.92 ± 0.22	0.91 ± 0.22	0.89 ± 0.20	0.87 ± 0.19	0.91 ± 0.22
LDL-C (mmol/l)*	2.90 ± 0.81	2.88 ± 0.79	2.90 ± 0.74	2.83 ± 0.78	2.90 ± 0.80
TG (mmol/l)†	2.08 (1.52, 2.69)	2.05 (1.41, 2.62)	2.10 (1.69, 2.71)	2.03 (1.59, 2.68)	1.92 (1.42, 2.53)

Please see list of abbreviations used. *mean ± SD are given for normally distributed variables, †median (25^th ^and 75^th ^percentile values) are given for variables with a skewed distribution ‡the frequency of men or women with the metabolic syndrome according to each definition.

**Table 5:** Comparison among subjects free of metabolic syndrome, WHO-defined metabolic syndrome and surplus IDF-defined metabolic syndrome.

Parameter	No-MS	WHO-MS	Surplus-MS (IDF)	ANOVA *p*
Frequency *(n)*	50% (790)	20% (314)	30% (464)	
Age (years)	42 ± 13	47 ± 12^a^	49 ± 13^b^	<0.001
BMI (kg/m^2^)	27 ± 4	33 ± 5^a^	31 ± 4^b,c^	<0.001
SBP (mmHg)	124 ± 19	145 ± 22^a^	142 ± 26^b^	<0.001
DBP(mmHg)	80 ± 11	92 ± 12^a^	90 ± 12^b,c^	<0.001
Glucose (mmol/l)	4.8 ± 0.4	5.4 ± 0.6^a^	5.0 ± 0.6^b,c^	<0.001
Log insulin (pmol/l)	1.60 ± 0.22	2.01 ± 0.13^a^	1.69 ± 0.17^b,c^	<0.001
Log HOMA-IR	0.07 ± 0.23	0.53 ± 0.14^a^	0.18 ± 0.18^b,c^	<0.001
Framingham risk score	1.10 ± 1.87	2.99 ± 4.64^a^	2.93 ± 4.54^b^	<0.001
Women:				
Frequency *(n)*	51% (525)	18% (191)	31% (320)	
Waist (cm)	86.0 ± 11.4	102.8 ± 9.7^a^	95.8 ± 9.5^b,c^	<0.001
TC (mmol/l)	4.64 ± 1.06	4.99 ± 1.13^a^	4.86 ± 1.00^b^	<0.001
HDL-C (mmol/l)	1.36 ± 0.31	1.07 ± 0.26^a^	1.09 ± 0.23^b^	<0.001
LDL-C (mmol/l)	2.82 ± 0.92	3.06 ± 0.97^a^	3.04 ± 0.85^b^	<0.001
Log TG (mmol/l)	-0.02 ± 0.16	0.23 ± 0.19^a^	0.16 ± 0.19^b,c^	<0.001
Men:				
Frequency *(n)*	50% (265)	23% (123)	27% (144)	
Waist (cm)	94.2 ± 9.8	107.0 ± 8.3^a^	103.1 ± 6.0^b,c^	<0.001
TC (mmol/l)	4.57 ± 0.93	4.89 ± 0.91^a^	4.79 ± 0.94	0.004
HDL-C (mmol/l)	1.08 ± 0.24	0.92 ± 0.22^a^	0.92 ± 0.24^b^	<0.001
LDL-C (mmol/l)	2.90 ± 0.82	2.90 ± 0.81	2.92 ± 0.84	0.957
Log TG (mmol/l)	0.07 ± 0.19	0.32 ± 0.23^a^	0.25 ± 0.22^b,c^	<0.001

Please see list of abbreviations used. Data is presented as mean ± SD. No-MS: subjects free of metabolic syndrome (WHO and IDF negative), WHO-MS: metabolic syndrome by WHO definition, including subjects identified concordantly by IDF (WHO positive, IDF either positive or negative), surplus-MS: subjects identified additionally as metabolic syndrome by only IDF definition (WHO negative, IDF positive).

a: p < 0.05 No-MS vs. WHO-MS, estimated by post hoc Tukey's test

b: p < 0.05 No-MS vs. surplus-MS (IDF), estimated by post hoc Tukey's test

c: p < 0.05 WHO-MS vs. surplus-MS (IDF), estimated by post hoc Tukey's test.

## Discussion

Our data show distinct differences in the prevalence of metabolic syndrome and the level of agreement between the definitions that do not require the measurement of insulin levels and the definitions that do require. The novel aspect of our study is that the agreement among all of these five published definitions has not been documented before and the analysis was conducted in a population with a high prevalence of metabolic syndrome. The variability in prevalence estimates reflects different cut-off points and different combinations of criteria among various definitions. The prevalence estimates of National Cholesterol Education Program Adult Treatment Panel III, American College of Endocrinology and International Diabetes Federation definitions are close to each other. ACE proposes to diagnose metabolic syndrome only in individuals who are at increased risk of insulin resistance. BMI over 25 kg/m^2^, sedentary lifestyle and a positive family history of cardiovascular disease are among prerequisites of ACE definition [11] and these are common occurrences in the population. Ninety-seven percent of our study population had one of the prerequisite conditions of ACE definition. ACE and IDF definitions proved to be almost identical in this study.

A health care provider has two alternative sets of metabolic syndrome definitions whether he or she decides to diagnose patients as having or not having the metabolic syndrome: a definition that require an insulin assay and a definition that do not require it. By restricting high risk patients (the denominator) to individuals with high insulin levels rather than the general population, WHO and EGIR criteria identifies only insulin resistant individuals with the metabolic syndrome and misses many individuals who are at increased risk of cardiovascular disease, but without elevated insulin levels. We aimed to characterize the subjects that are missed by the insulin measurement requiring definitions. In our sample, there were more subjects below the 75^th ^percentile of the HOMA-IR or insulin distribution, who had abdominal obesity, elevated blood pressure, low HDL-C levels, elevated TC/HDL-C ratio or elevated triglycerides. Metabolic syndrome components of low HDL-C and high blood pressure are classical risk factors for cardiovascular disease [1] and elevated triglyceride levels are shown to be a predictor of cardiovascular events in prospective studies in Turkey and elsewhere [31-33]. Abdominal obesity and elevated waist circumference has also been associated with increased CVD morbidity [34,35]. In view of our data, we conclude that both WHO and IDF definitions identify patients with insulin resistance and increased cardiovascular risks factors, but IDF definition identifies additional subjects, not identified by WHO definition and these additional subjects are at increased cardiovascular disease risk with lesser degree of insulin resistance. When we replaced EGIR for WHO definition and NCEP or ACE for IDF definition, we reached to the same conclusion.

### Relevant previous studies

San Antonio Heart Study, a prospective study with a median follow-up period of 7.4 years showed that Framingham Risk Scoring System was better in predicting future CVD events than the diagnosis of the metabolic syndrome and NCEP, IDF and WHO definitions imparted similar CVD and diabetes risks [5]. ACE and EGIR definitions were not evaluated in the San Antonio Heart Study. Hoorn Study, a prospective cohort study of diabetes and diabetes complications in the Dutch population compared NCEP, WHO, EGIR and ACE definitions with respect to their association with 10-year risk of fatal and nonfatal cardiovascular disease [4]. Hoorn Study Investigators reported that NCEP definition was associated with an approximately 2-fold risk of all end points in men and of nonfatal CVD in women after adjustment for age. The hazard ratios of the WHO, EGIR and ACE definitions for all end points were slightly lower. In the Hoorn Study, the kappa agreement between the definitions not requiring insulin measurement (NCEP or ACE) and the definitions requiring insulin measurement (WHO or EGIR) ranged from 0.28 to 0.46 and was fair to moderate, a similar outcome as our results. IDF definition was not evaluated by the Hoorn Study, because it was not available at that time. Guerrero-Romero and Rodriguez-Moran [22] found a very good agreement (κ: 0.87) between NCEP and IDF definitions and a moderate agreement (κ: 0.51) between IDF and WHO definitions. They concluded that IDF definition has a low concordance with WHO definition and a high concordance with NCEP definition and both NCEP and IDF definitions identify similar proportions of subjects with the metabolic syndrome. ACE and EGIR definitions were not analyzed in that report [22]. In the Korean Health and Examination Survey, the prevalence of the metabolic syndrome was 18.8% by NCEP and 19.5% by IDF definition. The agreement between NCEP and IDF definitions was moderate (κ: 0.54) [19]. The difference in the prevalence and agreement rates between the Korean Health and Examination Survey and our results, may probably be explained by the difference in the characteristics of the study populations. Our data and the results of other studies illustrate that the definitions with a similar design have a very good agreement [5,16,19-22].

### Limitations of the study

Our study is cross-sectional and compares CVD risk factors among metabolic syndrome definitions. There are only few prospective studies with IDF criteria [5,16,18,36-39], probably because it is a recently published definition. More prospective studies comparing IDF definition with others are needed. There have been no cross-sectional or prospective studies comparing all of the five published definitions of the metabolic syndrome, although there are some studies involving two to four of the definitions [4-7,14,16-24,36-39]. Another limitation of our study is that the insulin sensitivity is determined by HOMA-IR, a formula not perfectly correlated with the gold standard method, insulin sensitivity index derived from hyperinsulinemic euglycemic clamp. Studies show that the correlation between HOMA-IR value and the insulin sensitivity index range from 0.69 to 0.79 [40,41]. However, HOMA-IR calculation is more feasible for epidemiologic studies involving large number of participants. Microalbuminuria was not determined and not counted as a component in WHO definition in our study. Microalbuminuria is a predictor of future cardiovascular events [9,42]. In Insulin Resistance Atherosclerosis Study, only 6% of participants without diabetes had evidence of microalbuminuria [14]. Therefore, the effect of inclusion or exclusion of microalbuminuria in defining metabolic syndrome in subjects with normoglycemia appears minimal. The prevalence of impaired glucose tolerance was reported as 6.7% in the Turkish Diabetes Epidemiology Study (TURDEP), a nationwide population based cross-sectional study [43]. As we did not perform oral glucose tolerance testing, we may have underestimated the prevalence of metabolic syndrome, up to 6.7% for ACE, EGIR or WHO definitions. Some of the subjects with impaired glucose tolerance or microalbuminuria may nevertheless have been categorized as metabolic syndrome cases in our analyses, because they may have met other components of ACE, EGIR or WHO definitions.

Previous studies have shown widely varying estimates of the prevalence of metabolic syndrome, because of dissimilar definitions and populations: from 5% in Chinese men to 39% in Western populations [17,23]. Although our aim was not to document the prevalence of metabolic syndrome and our sample was not nationally representative, our results are in line with previous population based epidemiologic studies from Turkey, reporting a prevalence rate between 33 to 39% [44-46]. The prevalence of metabolic syndrome was reported as 37% in Greece [47], a neighbor country of Turkey. Although Turkey is a developing country, the age standardized cardiovascular death rate is estimated to rank among the highest in Europe [48]. The Turkish population has a cardiovascular disease risk factor profile that distinctly differs from that of Western populations, as the relative role of the metabolic syndrome and atherogenic dyslipidemia is more pronounced [48]. The leading independent predictors of CVD morbidity and mortality are related to the metabolic syndrome [45].

## Conclusion

National Cholesterol Education Program Adult Treatment Panel III, International Diabetes Federation and American College of Endocrinology definitions of the metabolic syndrome, the definitions that do not require the measurement of insulin levels have substantial to very good agreement and capture more individuals at increased cardiometabolic risk than the definitions that require insulin measurement. The agreement between World Health Organization and European Group for Study of Insulin Resistance definitions, the definitions that require the measurement of insulin sensitivity and fasting insulin levels is very good. In view of our data, we agree with NCEP, ACE and IDF and recommend the clinical use of definitions that do not require the measurement of insulin levels.

## List of abbreviations used

ACE: American College of Endocrinology, BMI: body mass index, BP: blood pressure, CVD: cardiovascular disease, DBP: diastolic blood pressure, DM: diabetes mellitus, EGIR: European Group for the Study of Insulin Resistance, HDL-C: high density lipoprotein cholesterol, HOMA-IR: homeostasis model assessment of insulin resistance, IDF: International Diabetes Federation, IFG: impaired fasting glucose, IGT: impaired glucose tolerance, IR: insulin resistance, LDL-C: low density lipoprotein cholesterol, MS: metabolic syndrome, NCEP: National Cholesterol Education Program, SBP: systolic blood pressure, SD: standard deviation, TC: total cholesterol, TG: triglyceride, WC: waist circumference, WHO: World Health Organization, WHR: waist to hip ratio.

## Competing interests

The author(s) declare that they have no competing interests.

## Authors' contributions

ASC participated in data acquisition, organization, and evaluation, statistical analysis, and wrote the manuscript. TPB participated in data acquisition and evaluation. All authors read and approved the final manuscript.

## Pre-publication history

The pre-publication history for this paper can be accessed here:



## Supplementary Material

Additional file 1 Explanation of subject recruitment. Click here for file

Additional file 2 Table 3a and Table 3b – Analysis of agreement among definitions of metabolic syndrome according to rural versus urban residence and gender. Click here for file

Additional file 3 Table 6 – Comparison among subjects free of the metabolic syndrome, WHO-defined metabolic syndrome and surplus NCEP-defined metabolic syndrome. Click here for file

Additional file 4 Table 7. Comparison among subjects free of the metabolic syndrome, WHO-defined metabolic syndrome and surplus ACE-defined metabolic syndrome. Click here for file

Additional file 5 Table 8. Comparison among subjects free of the metabolic syndrome, EGIR-defined metabolic syndrome and surplus IDF-defined metabolic syndrome. Click here for file

Additional file 6 Table 9. Comparison among subjects free of the metabolic syndrome, with EGIR-defined metabolic syndrome and surplus NCEP-defined metabolic syndrome. Click here for file

Additional file 7 Table 10. Comparison among subjects free of the metabolic syndrome, with EGIR-defined metabolic syndrome and surplus ACE-defined metabolic syndrome. Click here for file
