# Supplementary material for: Analysis of agreement among definitions of metabolic syndrome in nondiabetic Turkish adults: a methodological study
Source: BMC Public Health. 2007 Dec 19;7:353. doi: 10.1186/1471-2458-7-353 (PMC2249584; doi:10.1186/1471-2458-7-353)
Supplement: Additional file 1 — Explanation of subject recruitment. [file 1471-2458-7-353-S1.DOC]

**Subject recruitment:**

Subjects were invited to the study from the offices of local governors, the mukhtars. The mukhtar is the elected head of a village in rural areas or elected head of a neighborhood in towns or cities in Turkey. The mukhtar is personally known in the community. Address and identity information of the citizens are kept in the mukhtar’s office where two to three secretaries usually work. Fliers were posted and subjects were verbally informed of the study aims, procedures, informed consent and the fasting requirement by the study staff at the local governors’ offices. The mukhtar and his staff also informed the community about the study. One and two day prior to and on the day of phlebotomy, a verbal announcement of the study with above pertinent information was made through speakers of the minaret of local mosques. These verbal announcements were clearly audible from streets and inside the buildings in the community. No financial incentive was paid to the subjects. In summary, subjects self-selected themselves to participate.
