# Supplementary material for: Analysis of agreement among definitions of metabolic syndrome in nondiabetic Turkish adults: a methodological study
Source: BMC Public Health. 2007 Dec 19;7:353. doi: 10.1186/1471-2458-7-353 (PMC2249584; doi:10.1186/1471-2458-7-353)
Supplement: Additional file 2 — Table 3a and Table 3b – Analysis of agreement among definitions of metabolic syndrome according to rural versus urban residence and gender. [file 1471-2458-7-353-S2.DOC]

## Table 3a - Agreement among five definitions of the metabolic syndrome according to rural versus urban residence.

| Urban  Rural | NCEP | IDF | ACE | WHO | EGIR |
| --- | --- | --- | --- | --- | --- |
| NCEP | ***** | **0.84** | **0.75** | **0.43** | 0.43 |
| IDF | **0.84** | ***** | **0.79** | **0.39** | 0.40 |
| ACE | **0.80** | **0.84** | ***** | **0.38** | 0.40 |
| WHO | **0.26** | **0.24** | **0.22** | ***** | 0.84 |
| EGIR | 0.26 | 0.25 | 0.24 | 0.82 | * |

See list of abbreviations used. *n*= 1050 for urban, *n*=518 for rural residence.

## Table 3b - Agreement among five definitions of the metabolic syndrome according to gender.

| Men  Women | NCEP | IDF | ACE | WHO | EGIR |
| --- | --- | --- | --- | --- | --- |
| NCEP | ***** | **0.74** | **0.69** | **0.40** | 0.41 |
| IDF | **0.89** | ***** | **0.71** | **0.35** | 0.38 |
| ACE | **0.81** | **0.85** | ***** | **0.28** | 0.31 |
| WHO | **0.36** | **0.33** | **0.33** | ***** | 0.85 |
| EGIR | 0.35 | 0.33 | 0.35 | 0.82 | * |

See list of abbreviations used. *n*=532 for men, *n*=1036 for women.
