# Supplementary material for: Analysis of agreement among definitions of metabolic syndrome in nondiabetic Turkish adults: a methodological study
Source: BMC Public Health. 2007 Dec 19;7:353. doi: 10.1186/1471-2458-7-353 (PMC2249584; doi:10.1186/1471-2458-7-353)
Supplement: Additional file 3 — Table 6 – Comparison among subjects free of the metabolic syndrome, WHO-defined metabolic syndrome and surplus NCEP-defined metabolic syndrome. [file 1471-2458-7-353-S3.DOC]

## Table 6 - Comparison among subjects free of metabolic syndrome, WHO-defined metabolic syndrome and surplus NCEP-defined metabolic syndrome.

| Parameter | No-MS | WHO-MS | Surplus-MS (NCEP) | ANOVA *p* |
| --- | --- | --- | --- | --- |
| Frequency *(n)* | 54% (853) | 20% (314) | 26% (401) |  |
| Age (years) | 42±13 | 47±12^a^ | 49±13^b,c^ | <0.001 |
| BMI (kg/m^2^) | 27±4 | 33±5^a^ | 31±4^b,c^ | <0.001 |
| SBP (mmHg) | 124±19 | 145±22^a^ | 145±25^b^ | <0.001 |
| DBP(mmHg) | 80±11 | 92±12^a^ | 91±12^b^ | <0.001 |
| Glucose (mmol/l) | 4.8±0.4 | 5.4±0.6^a^ | 5.1±0.6^b,c^ | <0.001 |
| Log insulin (pmol/l) | 1.61±0.22 | 2.01±0.13^a^ | 1.68±0.17^b,c^ | <0.001 |
| Log HOMA-IR | 0.08±0.23 | 0.53±0.14^a^ | 0.17±0.18^b,c^ | <0.001 |
| Framingham risk score | 1.13±1.94 | 2.99±4.64^a^ | 3.16±4.73^b^ | <0.001 |
| Women: |  |  |  |  |
| Frequency *(n)* | 54% (564) | 19% (191) | 27% (281) |  |
| Waist (cm) | 85.9±11.0 | 102.8±9.7^a^ | 97.3±9.2^b,c^ | <0.001 |
| TC (mmol/l) | 4.63±1.04 | 4.99±1.13^a^ | 4.91±1.02^b^ | <0.001 |
| HDL-C (mmol/l) | 1.35±0.31 | 1.07±0.26^a^ | 1.08±0.22^b^ | <0.001 |
| LDL- C (mmol/l) | 2.80±0.90 | 3.06±0.97^a^ | 3.09±0.86^b^ | <0.001 |
| Log TG (mmol/l) | -0.02±0.16 | 0.23±0.19^a^ | 0.17±0.19^b,c^ | <0.001 |
| Men: |  |  |  |  |
| Frequency *(n)* | 54% (289) | 23% (123) | 23% (120) |  |
| Waist (cm) | 95.0±9.2 | 107.0±8.3^a^ | 102.9±8.3^b,c^ | <0.001 |
| TC (mmol/l) | 4.55±0.95 | 4.89±0.91^a^ | 4.88±0.86^b^ | <0.001 |
| HDL-C (mmol/l) | 1.08±0.24 | 0.92±0.22^a^ | 0.90±0.21^b^ | <0.001 |
| LDL- C (mmol/l) | 2.89±0.85 | 2.90±0.81 | 2.96±0.76 | 0.673 |
| Log TG (mmol/l) | 0.07±0.19 | 0.32±0.23^a^ | 0.29±0.22^b^ | <0.001 |

Please see list of abbreviations used. Data is presented as mean±SD. No-MS: subjects free of metabolic syndrome (WHO and NCEP negative), WHO-MS: metabolic syndrome by WHO definition, including subjects identified concordantly by NCEP (WHO positive, NCEP either positive or negative), surplus-MS: subjects identified additionally as metabolic syndrome by only NCEP definition (WHO negative, NCEP positive).

a: p<0.05 No-MS vs. WHO-MS, estimated by post hoc Tukey’s test

b: p<0.05 No-MS vs. surplus-MS (NCEP), estimated by post hoc Tukey’s test

c: p<0.05 WHO-MS vs. surplus-MS (NCEP), estimated by post hoc Tukey’s test.
