# Supplementary material for: Analysis of agreement among definitions of metabolic syndrome in nondiabetic Turkish adults: a methodological study
Source: BMC Public Health. 2007 Dec 19;7:353. doi: 10.1186/1471-2458-7-353 (PMC2249584; doi:10.1186/1471-2458-7-353)
Supplement: Additional file 4 — Table 7. Comparison among subjects free of the metabolic syndrome, WHO-defined metabolic syndrome and surplus ACE-defined metabolic syndrome. [file 1471-2458-7-353-S4.DOC]

## Table 7. Comparison among subjects free of metabolic syndrome, WHO-defined metabolic syndrome and surplus ACE-defined metabolic syndrome.

| Parameter | No-MS | WHO-MS | Surplus-MS (ACE) | ANOVA *p* |
| --- | --- | --- | --- | --- |
| Frequency *(n)* | 51% (804) | 20% (314) | 29% (450) |  |
| Age (years) | 42±13 | 47±12^a^ | 48±13^b^ | <0.001 |
| BMI (kg/m^2^) | 28±4 | 33±5^a^ | 30±4^b,c^ | <0.001 |
| SBP (mmHg) | 125±20 | 145±22^a^ | 142±25^b^ | <0.001 |
| DBP(mmHg) | 80±11 | 92±12^a^ | 90±12^b,c^ | <0.001 |
| Glucose (mmol/l) | 4.9±0.5 | 5.4±0.6^a^ | 5.0±0.5^b,c^ | <0.001 |
| Log insulin (pmol/l) | 1.61±0.22 | 2.01±0.13^a^ | 1.67±0.18^b,c^ | <0.001 |
| Log HOMA-IR | 0.09±0.23 | 0.53±0.14^a^ | 0.15±0.19^b,c^ | <0.001 |
| Framingham risk score | 1.14±1.96 | 2.99±4.64^a^ | 2.93±4.55^b^ | <0.001 |
| Women: |  |  |  |  |
| Frequency *(n)* | 53.4% (553) | 18.4% (191) | 28.2% (292) |  |
| Waist (cm) | 87.1±11.5 | 102.8±9.7^a^ | 94.7±10.5^b,c^ | <0.001 |
| TC (mmol/l) | 4.65±1.01 | 4.99±1.13^a^ | 4.86±1.08^b^ | <0.001 |
| HDL-C (mmol/l) | 1.37±0.30 | 1.07±0.26^a^ | 1.06±0.22^b^ | <0.001 |
| LDL- C (mmol/l) | 2.82±0.87 | 3.06±0.97^a^ | 3.04±0.93^b^ | <0.001 |
| Log TG (mmol/l) | -0.03±0.15 | 0.23±0.19^a^ | 0.18±0.19^b,c^ | <0.001 |
| Men: |  |  |  |  |
| Frequency *(n)* | 47% (251) | 23% (123) | 30% (158) |  |
| Waist (cm) | 95.8±10.0 | 107.0±8.3^a^ | 99.8±8.5^b,c^ | <0.001 |
| TC (mmol/l) | 4.60±0.96 | 4.89±0.91^a^ | 4.73±0.90 | 0.018 |
| HDL-C (mmol/l) | 1.12±0.24 | 0.92±0.22^a^ | 0.88±0.20^b^ | <0.001 |
| LDL- C (mmol/l) | 2.95±0.84 | 2.90±0.81 | 2.85±0.79 | 0.499 |
| Log TG (mmol/l) | 0.04±0.16 | 0.32±0.23^a^ | 0.29±0.22^b^ | <0.001 |

Please see list of abbreviations used. Data is presented as mean±SD. No-MS: subjects free of metabolic syndrome (WHO and ACE negative), WHO-MS: metabolic syndrome by WHO definition, including subjects identified concordantly by ACE (WHO positive, ACE either positive or negative), surplus-MS: subjects identified additionally as metabolic syndrome by only ACE definition (WHO negative, ACE positive).

a: p<0.05 No-MS vs. WHO-MS, estimated by post hoc Tukey’s test

b: p<0.05 No-MS vs. surplus-MS (ACE), estimated by post hoc Tukey’s test

c: p<0.05 WHO-MS vs. surplus-MS (ACE), estimated by post hoc Tukey’s test.
