# Supplementary material for: Analysis of agreement among definitions of metabolic syndrome in nondiabetic Turkish adults: a methodological study
Source: BMC Public Health. 2007 Dec 19;7:353. doi: 10.1186/1471-2458-7-353 (PMC2249584; doi:10.1186/1471-2458-7-353)
Supplement: Additional file 5 — Table 8. Comparison among subjects free of the metabolic syndrome, EGIR-defined metabolic syndrome and surplus IDF-defined metabolic syndrome. [file 1471-2458-7-353-S5.DOC]

## Table 8. Comparison among subjects free of metabolic syndrome, EGIR-defined metabolic syndrome and surplus IDF-defined metabolic syndrome.

| Parameter | No-MS | EGIR-MS | Surplus-MS (IDF) | ANOVA *p* |
| --- | --- | --- | --- | --- |
| Frequency *(n)* | 50% (787) | 21% (330) | 29% (451) |  |
| Age (years) | 42±13 | 46±12^a^ | 49±13^b,c^ | <0.001 |
| BMI (kg/m^2^) | 27±4 | 33±5^a^ | 31±4^b,c^ | <0.001 |
| SBP (mmHg) | 124±19 | 144±23^a^ | 143±26^b^ | <0.001 |
| DBP(mmHg) | 80±11 | 92±12^a^ | 90±12^b,c^ | <0.001 |
| Glucose (mmol/l) | 4.8±0.4 | 5.3±0.6^a^ | 5.1±0.6^b,c^ | <0.001 |
| Log insulin (pmol/l) | 1.60±0.21 | 2.01±0.13^a^ | 1.68±0.16^b,c^ | <0.001 |
| Log HOMA-IR | 0.07±0.23 | 0.53±0.15^a^ | 0.17±0.18^b,c^ | <0.001 |
| Framingham risk score | 1.10±1.87 | 2.79±4.45^a^ | 3.05±4.66^b^ | <0.001 |
| Women: |  |  |  |  |
| Frequency *(n)* | 50.5% (523) | 19% (197) | 30.5% (316) |  |
| Waist (cm) | 86.0±11.5 | 101.3±10.1^a^ | 96.4±9.8^b,c^ | <0.001 |
| TC (mmol/l) | 4.64±1.05 | 4.94±1.13^a^ | 4.89±1.00^b^ | <0.001 |
| HDL-C (mmol/l) | 1.36±0.31 | 1.06±0.27^a^ | 1.10±0.23^b^ | <0.001 |
| LDL- C (mmol/l) | 2.81±0.91 | 3.04±0.96^a^ | 3.05±0.85^b^ | <0.001 |
| Log TG (mmol/l) | -0.02±0.16 | 0.22±0.19^a^ | 0.16±0.19^b,c^ | <0.001 |
| Men: |  |  |  |  |
| Frequency *(n)* | 50% (264) | 25% (133) | 25% (135) |  |
| Waist (cm) | 94.0±9.7 | 106.9±8.1^a^ | 103.1±6.0^b,c^ | <0.001 |
| TC (mmol/l) | 4.58±0.93 | 4.85±0.90^a^ | 4.80±0.96 | 0.011 |
| HDL-C (mmol/l) | 1.09±0.24 | 0.91±0.22^a^ | 0.93±0.23^b^ | <0.001 |
| LDL- C (mmol/l) | 2.90±0.82 | 2.88±0.79 | 2.94±0.86 | 0.794 |
| Log TG (mmol/l) | 0.07±0.19 | 0.31±0.24^a^ | 0.25±0.21^b^ | <0.001 |

Please see list of abbreviations used. Data is presented as mean±SD. No-MS: subjects free of metabolic syndrome (EGIR and IDF negative), EGIR-MS: metabolic syndrome by EGIR definition, including subjects identified concordantly by IDF (EGIR positive, IDF either positive or negative), surplus-MS: subjects identified additionally as metabolic syndrome by only IDF definition (EGIR negative, IDF positive).

a: p<0.05 No-MS vs. EGIR-MS, estimated by post hoc Tukey’s test

b: p<0.05 No-MS vs. surplus-MS (IDF), estimated by post hoc Tukey’s test

c: p<0.05 EGIR-MS vs. surplus-MS (IDF), estimated by post hoc Tukey’s test.
