# Supplementary material for: Analysis of agreement among definitions of metabolic syndrome in nondiabetic Turkish adults: a methodological study
Source: BMC Public Health. 2007 Dec 19;7:353. doi: 10.1186/1471-2458-7-353 (PMC2249584; doi:10.1186/1471-2458-7-353)
Supplement: Additional file 6 — Table 9. Comparison among subjects free of the metabolic syndrome, with EGIR-defined metabolic syndrome and surplus NCEP-defined metabolic syndrome. [file 1471-2458-7-353-S6.DOC]

## Table 9. Comparison among subjects free of metabolic syndrome, with EGIR-defined metabolic syndrome and surplus NCEP-defined metabolic syndrome.

| Parameter | No-MS | EGIR-MS | Surplus-MS (NCEP) | ANOVA *p* |
| --- | --- | --- | --- | --- |
| Frequency *(n)* | 54% (843) | 21% (330) | 25% (395) |  |
| Age (years) | 42±13 | 46±12^a^ | 50±13^b,c^ | <0.001 |
| BMI (kg/m^2^) | 27±4 | 33±5^a^ | 31±4^b,c^ | <0.001 |
| SBP (mmHg) | 124±19 | 144±23^a^ | 145±26^b^ | <0.001 |
| DBP(mmHg) | 80±11 | 92±12^a^ | 91±12^b^ | <0.001 |
| Glucose (mmol/l) | 4.8±0.4 | 5.3±0.6^a^ | 5.1±0.6^b,c^ | <0.001 |
| Log insulin (pmol/l) | 1.60±0.21 | 2.01±0.13^a^ | 1.67±0.17^b,c^ | <0.001 |
| Log HOMA-IR | 0.08±0.23 | 0.53±0.15^a^ | 0.17±0.18^b,c^ | <0.001 |
| Framingham risk score | 1.13±1.95 | 2.79±4.45^a^ | 3.28±4.83^b^ | <0.001 |
| Women: |  |  |  |  |
| Frequency *(n)* | 54% (558) | 19% (197) | 27% (281) |  |
| Waist (cm) | 86.0±11.1 | 101.3±10.1^a^ | 97.9±9.5^b,c^ | <0.001 |
| TC (mmol/l) | 4.63±1.03 | 4.94±1.13^a^ | 4.93±1.02^b^ | <0.001 |
| HDL-C (mmol/l) | 1.35±0.31 | 1.06±0.27^a^ | 1.09±0.22^b^ | <0.001 |
| LDL- C (mmol/l) | 2.80±0.90 | 3.04±0.96^a^ | 3.10±0.87^b^ | <0.001 |
| Log TG (mmol/l) | -0.02±0.16 | 0.22±0.19^a^ | 0.17±0.19^b,c^ | <0.001 |
| Men: |  |  |  |  |
| Frequency *(n)* | 54% (285) | 25% (133) | 21% (114) |  |
| Waist (cm) | 94.9±9.1 | 106.9±8.1^a^ | 102.7±8.5^b,c^ | <0.001 |
| TC (mmol/l) | 4.57±0.95 | 4.85±0.90^a^ | 4.88±0.88^b^ | 0.001 |
| HDL-C (mmol/l) | 1.08±0.25 | 0.91±0.22^a^ | 0.90±0.21^b^ | <0.001 |
| LDL- C (mmol/l) | 2.90±0.85 | 2.88±0.79 | 2.96±0.78 | 0.695 |
| Log TG (mmol/l) | 0.06±0.18 | 0.31±0.24^a^ | 0.30±0.21^b^ | <0.001 |

Please see list of abbreviations used. Data is presented as mean±SD. No-MS: subjects free of metabolic syndrome (EGIR and NCEP negative), EGIR-MS: metabolic syndrome by EGIR definition, including subjects identified concordantly by NCEP (EGIR positive, NCEP either positive or negative), surplus-MS: subjects identified additionally as metabolic syndrome by only NCEP definition (EGIR negative, NCEP positive).

a: p<0.05 No-MS vs. EGIR-MS, estimated by post hoc Tukey’s test

b: p<0.05 No-MS vs. surplus-MS (NCEP), estimated by post hoc Tukey’s test

c: p<0.05 EGIR-MS vs. surplus-MS (NCEP), estimated by post hoc Tukey’s test.
