# Supplementary material for: Analysis of agreement among definitions of metabolic syndrome in nondiabetic Turkish adults: a methodological study
Source: BMC Public Health. 2007 Dec 19;7:353. doi: 10.1186/1471-2458-7-353 (PMC2249584; doi:10.1186/1471-2458-7-353)
Supplement: Additional file 7 — Table 10. Comparison among subjects free of the metabolic syndrome, with EGIR-defined metabolic syndrome and surplus ACE-defined metabolic syndrome. [file 1471-2458-7-353-S7.DOC]

## Table 10. Comparison among subjects free of metabolic syndrome, with EGIR-defined metabolic syndrome and surplus ACE-defined metabolic syndrome.

| Parameter | No-MS | EGIR-MS | Surplus-MS (ACE) | ANOVA *p* |
| --- | --- | --- | --- | --- |
| Frequency *(n)* | 51% (803) | 21% (330) | 28% (435) |  |
| Age (years) | 42±13 | 46±12^a^ | 49±13^b,c^ | <0.001 |
| BMI (kg/m^2^) | 28±4 | 33±5^a^ | 30±4^b,c^ | <0.001 |
| SBP (mmHg) | 125±20 | 144±23^a^ | 143±25^b^ | <0.001 |
| DBP(mmHg) | 80±11 | 92±12^a^ | 90±12^b^ | <0.001 |
| Glucose (mmol/l) | 4.9±0.5 | 5.3±0.6^a^ | 5.0±0.6^b,c^ | <0.001 |
| Log insulin (pmol/l) | 1.61±0.21 | 2.01±0.13^a^ | 1.66±0.17^b,c^ | <0.001 |
| Log HOMA-IR | 0.09±0.23 | 0.53±0.15^a^ | 0.15±0.18^b,c^ | <0.001 |
| Framingham risk score | 1.14±1.96 | 2.79±4.45^a^ | 3.07±4.68^b^ | <0.001 |
| Women: |  |  |  |  |
| Frequency *(n)* | 53% (553) | 19% (197) | 28% (286) |  |
| Waist (cm) | 87.3±11.8 | 101.3±10.1^a^ | 95.1±10.7^b,c^ | <0.001 |
| TC (mmol/l) | 4.65±1.00 | 4.94±1.13^a^ | 4.90±1.08^b^ | <0.001 |
| HDL-C (mmol/l) | 1.37±0.30 | 1.06±0.27^a^ | 1.07±0.22^b^ | <0.001 |
| LDL- C (mmol/l) | 2.82±0.87 | 3.04±0.96^a^ | 3.06±0.93^b^ | <0.001 |
| Log TG (mmol/l) | -0.03±0.15 | 0.22±0.19^a^ | 0.18±0.19^b,c^ | <0.001 |
| Men: |  |  |  |  |
| Frequency *(n)* | 47% (250) | 25% (133) | 28% (149) |  |
| Waist (cm) | 95.5±9.8 | 106.9±8.1^a^ | 99.9±8.7^b,c^ | <0.001 |
| TC (mmol/l) | 4.61±0.96 | 4.85±0.90 | 4.73±0.91 | 0.060 |
| HDL-C (mmol/l) | 1.12±0.24 | 0.91±0.22^a^ | 0.89±0.20^b^ | <0.001 |
| LDL- C (mmol/l) | 2.95±0.85 | 2.88±0.79 | 2.85±0.80 | 0.438 |
| Log TG (mmol/l) | 0.04±0.16 | 0.31±0.24^a^ | 0.28±0.21^b^ | <0.001 |

Please see list of abbreviations used. Data is presented as mean±SD. No-MS: subjects free of metabolic syndrome (EGIR and ACE negative), EGIR-MS: metabolic syndrome by EGIR definition, including subjects identified concordantly by ACE (EGIR positive, ACE either positive or negative), surplus-MS: subjects identified additionally as metabolic syndrome by only ACE definition (EGIR negative, ACE positive).

a: p<0.05 No-MS vs. EGIR-MS, estimated by post hoc Tukey’s test

b: p<0.05 No-MS vs. surplus-MS (ACE), estimated by post hoc Tukey’s test

c: p<0.05 EGIR-MS vs. surplus-MS (ACE), estimated by post hoc Tukey’s test.
